# Ankylosing spondylitis mimicking myocardial infarction: A diagnostic challenge, can be tricky to diagnose

**DOI:** 10.12669/pjms.41.9.11243

**Published:** 2025-09

**Authors:** Shaker Hassan S Alshehri, Mohamed Mohiuddin Malabari, Ali Ibrahim Alhefzi, Ahmed Onayzan Alshammari, Saadeldin Ahmed Idris

**Affiliations:** 1 Shaker Hassan S Alshehri, Department of Orthopedic Surgery, College of Medicine, King Khalid University, Abha, Saudi Arabia; 2 Mohamed Mohiuddin Malabari, Department of Adult and Pediatric Spine Surgery, Saudi German Hospital, Jeddah, Saudi Arabia; 3 Ali Ibrahim Alhefzi, Department of Orthopedic Surgery, College of Medicine, King Khalid University, Abha, Saudi Arabia; 4 Ahmed Onayzan Alshammari, Department of Orthopedic, College of Medicine, University of Hail, Hail, Saudi Arabia; 5 Saadeldin Ahmed Idris, Department of Surgery, College of Medicine, University of Hail, Hail, Saudi Arabia

**Keywords:** Ankylosing, Case Report, Diagnostic Challenge, Myocardial infarction, Osteoarthritis, Radiating back pain, Spondylitis, Vertebrae

## Abstract

The diagnosis of ankylosing spondylitis can be challenging when it mimics myocardial infarction symptoms. This case report describes a 30-year-old female patient who primarily presented with mid-back pain that radiated to her shoulder, bringing up the possibility of silent myocardial infarction. Additional investigations confirmed the existence of ankylosing spondylitis. The proximity of the vertebral column to vital organs might complicate such a diagnosis. This case report emphasizes the need to consider ankylosing spondylitis as a differential diagnosis in patients with odd symptoms resembling myocardial infarction, especially where the risk factors or characteristic signs are present. Early recognition of ankylosing spondylitis can assist in timely management and prevent unnecessary interventions.

## INTRODUCTION

Ankylosing spondylitis (AS) is a chronic inflammatory disease that primarily affects the axial skeleton, characterized by inflammatory back pain, sacroiliitis and limited spinal mobility. Nevertheless, it can present a wide range of clinical manifestations, making diagnosis difficult and potentially misdiagnosed. A particular diagnostic challenge comes to light when AS resembles myocardial infarction (MI), demonstrating a clinical dilemma for healthcare providers. On the other hand, cases of AS having MI have been widely mentioned in the literature.^1^ Because these are two separate diseases with distinct characteristics, risk assessment should be tailored to each individual.^2^

Due to the vertebral column’s proximity to vital organs, including the heart, it can be challenging to distinguish between AS and MI.^3^ Overlapping symptoms of chest and shoulder pain may complicate diagnosis, potentially leading to delays, misdiagnoses and inappropriate management.^1^ This case report highlights the importance that in patients with atypical symptoms resembling MI, particularly in the presence of pertinent risk factors or clinical signs, AS should be considered a differential diagnosis.

## CASE PRESENTATION

A 30-year-old female patient, diagnosed with Type-II diabetes and currently on oral hypoglycemic agents, presented to the Emergency Department with complaints of mid-back pain radiating to both sides of her chest and shoulders, accompanied by dyspnea. Her pain became more severe, prompting her to seek medical help. An electrocardiogram (ECG) and measurement of troponin level were conducted to rule out Acute Coronary Syndrome, which yielded a normal echocardiogram and troponin level. Despite these findings, she was diagnosed with a silent myocardial infarction. Further evaluations included laboratory tests and radiological imaging, after which she was referred to the general surgery department, where an abdominal ultrasound was undertaken that ruled out gallbladder disease and thus discharged home on analgesics.

**Fig.1:**
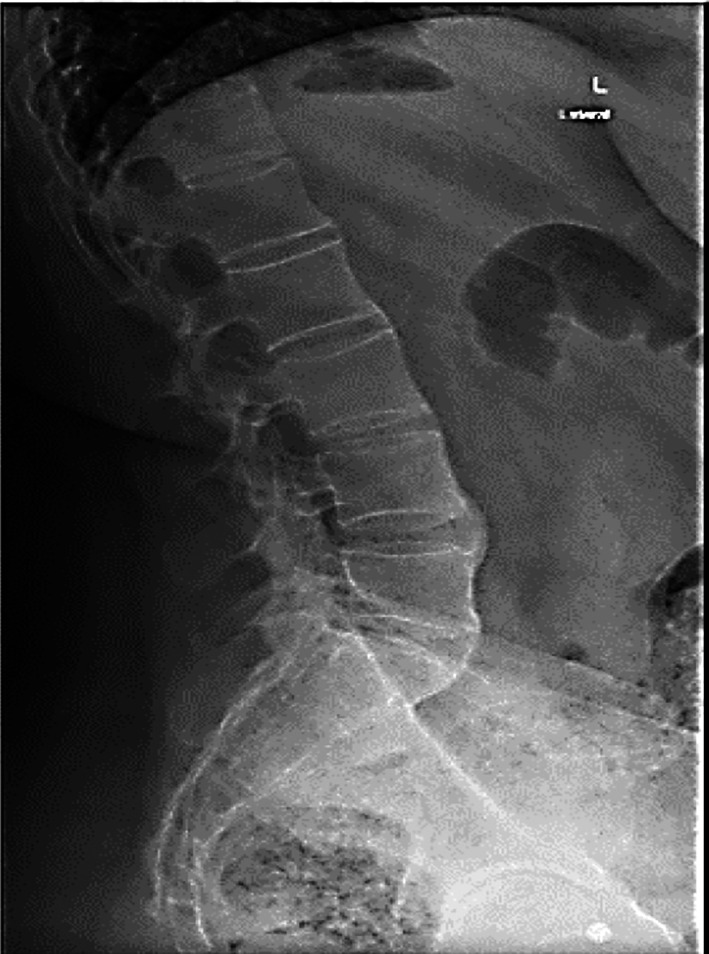
X-ray image illustrating paravertebral ossification in the form of syndesmophytes in a patient diagnosed with ankylosing spondylitis.

**Fig.2:**
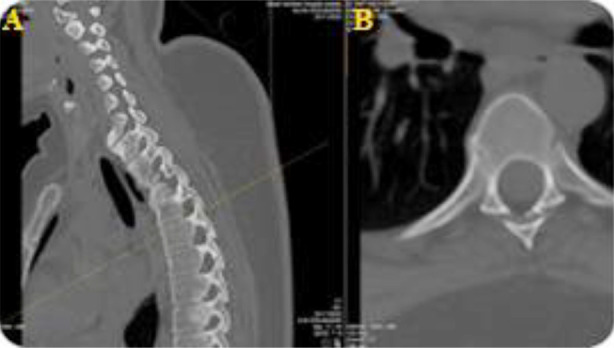
CT scan image demonstrating an Anderson lesion in a patient diagnosed with ankylosing spondylitis. **A:** Sagittal plane, **B:** Axial plane

**Fig.3:**
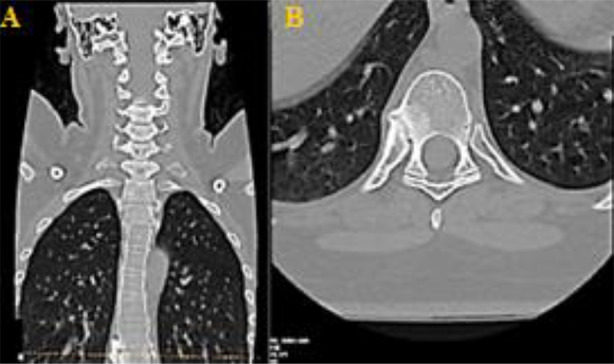
CT scan image illustrating costovertebral arthritis in a patient diagnosed with ankylosing spondylitis. **A:** Coronal plane, **B:** Axial plane.

**Fig.4:**
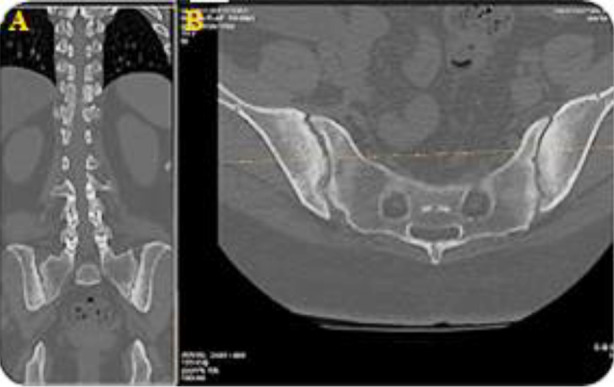
CT scan image depicting sacroiliitis in a patient with ankylosing spondylitis. A; coronal plane, B; Axial plane.

**Fig.5:**
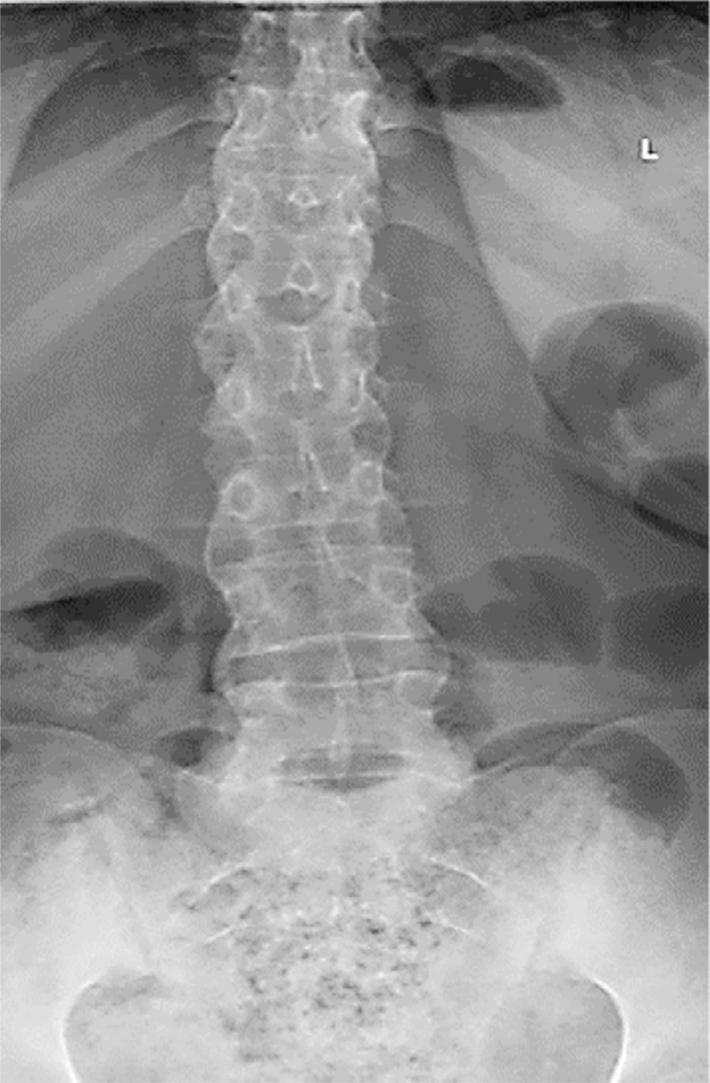
AP X-ray image demonstrating the characteristic bamboo appearance of the spine in a patient diagnosed with ankylosing spondylitis.

Subsequently, she visited the spine clinic with similar symptoms, where her medical history assessment revealed that she lost her mother at a young age due to unknown causes, recurrent eye irritation, urinary tract infections and gynecological issues, along with morning stiffness and hand pain. Physical examination revealed severe tenderness in the thoracic region, limited chest expansion, bilateral lower limb hypertonia (+1) and hyperreflexia. The Shober test was positive. A review of her medical records indicated paravertebral ossification and syndesmophytes from a prior X-ray at the Orthopedic clinic, suggesting a potential diagnosis of AS (Fig.1). Subsequently, a CT scan was performed, which revealed an Anderson lesion with bridging syndesmophytes linking adjacent vertebrae, leading to spinal segment fusion, along with costovertebral arthritis and sacroiliitis (Fig. 2, 3 and 4). Additionally, the X-ray scan indicated the characteristic appearance of a bamboo spine and the loss of intervertebral disc spaces (Fig.5).

Additional investigations yielded the following findings: the patient’s Rheumatoid Factor (RF) level was 20 IU/ml (within normal range). The HLA-B27 test returned positive, supporting the suspicion of ankylosing spondylitis (AS). The C-reactive protein (CRP) level was elevated at 33 mg/dL, raising concerns about AS. Conversely, the Antinuclear Antibody (ANA) test was negative, indicating no specific autoantibodies related to autoimmune disorders. The Erythrocyte Sedimentation Rate (ESR) was slightly elevated at 35 mm/hour, suggesting inflammation consistent with the diagnosis. Additionally, the Troponin test was negative, ruling out cardiac involvement. She was subsequently referred to the Rheumatology department, where treatment with certolizumab pegol, a tumor necrosis factor (TNF-α) inhibitor, significantly controlled symptoms and overall improved her condition.

### Ethics statement:

Written consent was obtained from the patient, accepting permission for the procedure and publication of images or data.

## DISCUSSION

Diagnosing AS can be difficult because many different diseases may present with identical symptoms, increasing the possibility of misdiagnosis. Delays in diagnosis can significantly impact multiple aspects of a patient’s life. In some individuals, particularly women, early symptoms of AS may manifest atypically, complicating diagnosis.^4^ This was evident in our case. While prompt and accurate diagnosis of AS is essential, it is often overlooked due to the absence of distinctive symptoms and definitive laboratory tests.^5^ The patient here had MI-mimicking symptoms, but AS was eventually diagnosed based on clinical observations, radiographic evidence and laboratory tests. Morning exacerbation of mid-back pain that was relieved by exercise, had raised suspicion for AS.

Imaging studies, including X-rays and CT scans, revealed characteristic features such as sacroiliitis, syndesmophytes anderson lesions, bamboo spine and costovertebral arthritis, aligning with the criteria established by Rudwaleit et al. in the Assessment of Spondyloarthritis International Society (ASAS).^6^ While laboratory results in AS are generally nonspecific, elevated acute-phase reactants like ESR and CRP are found in about 70% of patients with active disease,^7^ supporting the diagnosis alongside positive HLA-B27 testing.^8^ While the modified New York criteria do not mandate HLA-B27 for AS diagnosis, further research indicates that HLA-B27 exhibits significant sensitivity, specificity and a favorable likelihood ratio in evaluating AS patients.^5^

Furthermore, the diagnosis of As was established by the detection of the HLA-B27 indicator, which was in line with others who stated that approximately 80-95% of individuals diagnosed with AS exhibit the presence of the HLA-B27 marker.^9^ Clinical assessment can reveal sacroiliac joint pain, which can be evaluated through the Gaenslen or Patrick’s Faber test.^10^ In mild ankylosing spondylitis, imaging may show subtle changes and minimal sacroiliitis, with many individuals asymptomatic or experiencing only mild discomfort; radiological sacroiliitis indicates chronicity.^6^ In this case, the Faber test was positive. After the diagnosis of AS was confirmed, treatment was initiated, which included a multidisciplinary approach with physical therapy, NSAIDs and TNF-α.

Extensive research has convincingly demonstrated the rapid efficacy of TNF-α in treating multiple aspects of AS. These agents significantly improve clinical symptoms, although TNF-α tends to be less effective in women compared to men with AS. They also enhance physical performance, increase spinal mobility, alleviate peripheral joint disease and tendonitis and reduce acute reactions associated with AS.^8^

## CONCLUSION

Considering such a complicated case of a patient suffering from diabetes presenting with varied symptoms, proper deliberation and liaison among different medical specialties play a vital role in ascertaining the causative agent and thus forming an approachable strategy. Symptoms of AS may overlap with those of other disorders, as prompt recognition and diagnosis of AS is crucial to initiating appropriate treatment early and improving patient outcomes.

Timely treatment not only relieves pain but also helps prevent long-term sequelae. Clinicians should be vigilant and consider ankylosing spondylitis in patients with unexplained back pain, especially in younger patients. A comprehensive evaluation, including a patient history and imaging studies, can facilitate an accurate diagnosis.

## Data Availability

Data supporting the study results can be provided, following a request sent to the corresponding author’s email.

## AI usage:

The authors confirm that AI was not used in the preparation of this manuscript.

## Manuscript Revision:

The authors affirm that they have reviewed and revised the updated version of the manuscript.
